# Estrogenic Responsiveness of Brown Trout Primary Hepatocyte Spheroids to Environmental Levels of 17α-Ethinylestradiol

**DOI:** 10.3390/jox14030060

**Published:** 2024-08-06

**Authors:** Rodrigo F. Alves, Célia Lopes, Eduardo Rocha, Tânia Vieira Madureira

**Affiliations:** 1 Team of Animal Morphology and Toxicology, Interdisciplinary Centre of Marine and Environmental Research (CIIMAR/CIMAR), University of Porto (U.Porto), Terminal de Cruzeiros do Porto de Leixões, Av. General Norton de Matos s/n, 4450-208 Matosinhos, Portugal; rodrigo.r.f.alves@hotmail.com (R.F.A.); cclopes@icbas.up.pt (C.L.); erocha@icbas.up.pt (E.R.); 2 Laboratory of Histology and Embryology, Department of Microscopy, ICBAS—School of Medicine and Biomedical Sciences, University of Porto (U.Porto), Rua Jorge Viterbo Ferreira 228, 4050-313 Porto, Portugal

**Keywords:** brown trout, primary hepatocytes spheroids, environmental concentrations, 17α-ethinylestradiol, 3D cultures

## Abstract

Three-dimensional (3D) fish hepatocyte cultures are promising alternative models for replicating in vivo data. Few studies have attempted to characterise the structure and function of fish 3D liver models and illustrate their applicability. This study aimed to further characterise a previously established spheroid model obtained from juvenile brown trout (*Salmo trutta*) primary hepatocytes under estrogenic stimulation. The spheroids were exposed for six days to environmentally relevant concentrations of 17α-ethinylestradiol—EE2 (1–100 ng/L). The mRNA levels of peroxisome (*catalase—Cat* and *urate oxidase—Uox*), lipid metabolism (*acyl-CoA long chain synthetase 1—Acsl1*, *apolipoprotein AI—ApoAI*, and *fatty acid binding protein 1—Fabp1*), and estrogen-related (*estrogen receptor α—ERα*, *estrogen receptor β—ERβ*, *vitellogenin A—VtgA*, *zona pellucida glycoprotein 2.5—ZP2.5*, and *zona pellucida glycoprotein 3a.2—ZP3a.2*) target genes were evaluated by quantitative real-time polymerase chain reaction. Immunohistochemistry was used to assess Vtg and ZP protein expressions. At the highest EE2 concentration, *VtgA* and *ZP2.5* genes were significantly upregulated. The remaining target genes were not significantly altered by EE2. Vtg and ZP immunostaining was consistently increased in spheroids exposed to 50 and 100 ng/L of EE2, whereas lower EE2 levels resulted in a weaker signal. EE2 did not induce significant changes in the spheroids’ viability and morphological parameters. This study identified EE2 effects at environmentally relevant doses in trout liver spheroids, indicating its usefulness as a proxy for in vivo impacts of xenoestrogens.

## 1. Introduction

A wide range of negative impacts on aquatic species has been claimed by endocrine-disrupting chemicals (EDCs), namely estrogenic compounds [1]. In fish, estrogens may cause changes in lipid and steroid hormone profiles [2], disruptions in the biotransformation metabolism [2,3]), and even behavioural and body phenotype alterations [4]. In salmonids, the liver effects associated with the classic endocrine disruptor 17α-ethinylestradiol (EE2) have been intensely discussed and investigated using both in vivo [5,6,7] and in vitro [8,9,10,11] models. Transcriptomics has often been explored to study the impacts of EE2, as reviewed by Martyniuk et al. [12], unveiling estrogen-regulated genes. As an example, according to Hultman et al. [9], the microarray analysis of rainbow trout (*Oncorhynchus mykiss*) primary hepatocytes after EE2 exposure revealed gene expression alterations in distinct biological pathways related to estrogen receptor (ER) regulation, biotransformation, lipid metabolism, and cellular growth. In accordance, the EE2 treatment of liver slices of Atlantic cod (*Gadus morhua*) upregulated vitellogenin (*Vtg*) and zona pellucida (*ZP*) genes [13], both recognized as estrogenic biomarkers [14,15].

In mammals, three-dimensional (3D) hepatic models are increasingly being used as alternatives to in vivo assays for investigating metabolism [16,17], hepatic diseases [18], and hepatotoxicity [19], and even for testing new drug candidates [20].

While research on 3D systems in fish is not as advanced as that in mammals, studies in recent years have contributed to the establishment of experimental approaches for the maintenance of viable and functional hepatocytes from a variety of fish species [21,22,23,24,25]. There is an enormous potential for using 3D liver models in the context of environmental toxicology. In this line, spheroids from a clearfin livebearer (*Poeciliopsis lucida*) hepatocellular carcinoma (PLHC-1) cell line were used to test the effects of a mixture of plastic additives (1 to 50 µM) and revealed a liver-like lipid profile phenotype [23]. Further, PLHC-1 spheroids exposed to benzo(a)pyrene (1 nM to 5 μM) showed *Cyp1a* induction and cellular alterations, evidencing its high potential for screening the induction of xenobiotic metabolism and tissue damage [26].

Estrogenic compounds were already tested in fish hepatocyte spheroids. For example, Park et al. [25] found upregulations of *Vtg5* mRNA expressions in zebrafish liver (ZFL) cell line spheroids stimulated with 1 nM of 17β-estradiol (E2) for 24 h. Additionally, an increase in *ER* and *Vtg* mRNA levels was observed in rainbow trout hepatocyte spheroids from day 1 to day 8 in culture after 24 h exposure to 1 µM of E2 [27].

Primary hepatocyte brown trout spheroids were previously characterized in terms of viability, morphological parameters, histomorphology, and basal gene expression [21,22]. The present study aimed to test the applicability of those spheroids by exposing them to environmentally relevant concentrations of EE2 (1 to 100 ng/L). Gene expression analyses of a selection of target genes and immunohistochemistry were used to assess effects. To our knowledge, this is the first study using 3D cultures of fish primary hepatocytes under environmentally relevant concentrations of EE2. It uncovers innovative findings and sets baselines to enable further advances for implementing 3D cultures in fish toxicity assays.

## 2. Materials and Methods

### 2.1. Fish

A stock of sexually immature brown trout (*Salmo trutta*) of 12 (±2) months—mean (±SD)—was obtained from the Aquaculture Station of Torno (Amarante, Portugal). The animals were randomly distributed (maximum of 5 fish per tank) in 6 independent 100 L fiberglass tanks under a continuous dechlorinated water recirculation system and acclimatized for at least two weeks before use in diverse experiments. For this study, 6 animals were sampled from the different tanks. The fish were maintained under a photoperiod of 12 h light/12 h dark and fed daily ad libitum (Trout Plus 4, AquaSoja), except before isolations. The fish were visually inspected daily and did not have lack of appetite or any common stress behaviours. Finally, no gross lesions were noted, either when living or after sacrifice. Fish used in this study had a mean weight of 49.0 (±26.1) g and a mean total length of 16.3 (±2.9) cm.

The water physicochemical parameters were analysed at least once a week using commercial test kits to measure ammonium/ammonia and nitrates/nitrites (Prodac, Cittadella, Italy): ammonium and ammonia—0.0 (±0.0) mg/L, nitrates—35.4 (±4.2) mg/L, and nitrites—0.05 (±0.02) mg/L. Water temperature—19.3 (±1.4) °C and oxygen (O_2_)—90.1 (±1.5)% were determined using a portable instrument (DO210, VWR International, Leuven, Belgium), and pH levels—7.9 (±0.3) were measured using a pH reader (WTW pH530, Oberbayern, Germany).

### 2.2. Hepatocyte Isolation

Six fish were independently used for primary hepatocyte isolation. The fish were euthanized with an aqueous solution (0.6 mL/L) of ethylene glycol monophenyl ether (Merck KGaA, Darmstadt, Germany). All experimental procedures followed the Portuguese Decree-Law No. 113/2013, implementing EU Directive No. 2010/63 on animal protection for scientific purposes and respecting animal handling. The blood (±1 mL) was collected using insulin syringes through the caudal vein in order to minimize the amount of blood in the liver. After the liver was sampled, primary hepatocytes were isolated by ex situ collagenase perfusion using Hanks’ Balanced Salt Solution (HBSS), as previously described in detail [11]. Cell viability was checked in all experiments using an automatic cell counter (Invitrogen^TM^, Countess^TM^ Automated Cell Counter) based on the trypan blue exclusion assay; cell viability was 76% (±7.0).

### 2.3. Exposure Assays

Hepatocytes were cultured on four plates per fish at a cell density of 5 × 10^5^ cells/mL (total volume of 3 mL/well), using 6-well non-tissue culture treated sterile plates with flat bottom and low evaporation lid (351146, Falcon, Corning, New York, NY, USA). The culture medium consisted of Dulbecco’s modified Eagle medium/nutrient mixture F-12 (DMEM/F-12) (GE Healthcare Life Sciences, South Logan, UT, USA) with 10% fetal bovine serum (FBS) (Merck KGaA, Darmstadt, Germany), 15 mM of 2-[4-(2hydroxyethyl)1-piperazinyl]-ethanesulfonic acid (HEPES) (Merck KGaA, Darmstadt, Germany), and 10 mL/L of antibiotic/antimycotic solution (Merck KGaA, Darmstadt, Germany), since it has already proven to be ideal for cultures of brown trout primary hepatocyte spheroids (PHS) [21,22]. The culture medium (1.5 mL/well) was changed every 48 h. Hepatocytes were maintained at 18 °C, without an additional supply of O_2_/CO_2_, and at constant orbital agitation (~100 rpm) (IKA^®^ MTS 2/4 digital microtiter shaker, Higashiosaka, Japan). At the 12th day post-isolation, the spheroids were exposed (on the same initial plates) until the 18th day post-isolation to different conditions: control—C (DMEM/F-12 with 15 mM HEPES, 10 mL/L of antibiotic/antimycotic solution, and charcoal-stripped FBS 10%, *v*/*v*), solvent control—SC (0.1% ethanol in supplemented DMEM/F-12 medium), and four EE2 concentrations, 1—1 ng/L (0.003 nM), 10—10 ng/L (0.033 nM), 50—50 ng/L (0.169 nM), and 100—100 ng/L (0.337 nM) in supplemented DMEM/F-12 medium. Medium changes were performed on alternate days (total volume changed on the 14th and 16th days post-isolation), as previously established [21]. On the 18th day post-isolation, spheroids generated from each independent fish were sampled for cell viability (lactate dehydrogenase—LDH and resazurin assays), morphological parameters (area, equivalent diameter and sphericity), morphology, and molecular analyses.

### 2.4. Spheroids Morphological Parameters

For each experiment on the 18th day post-isolation, brown trout PHS were photographed (*n* = 30 spheroids/condition) under a 10× objective lens in an Olympus CKX41 light microscope connected to an M5C-CYL-PL-D685CU Pixelink^®^ camera (Barrington, NJ, USA). Spheroid photos were analysed using AnaSP software version 2.0 to obtain the area, equivalent diameter, and sphericity [28].

### 2.5. Lactate Dehydrogenase (LDH) Assay

An LDH Cytotoxicity WST Assay Kit (ENZ-KIT157, Enzo Life Sciences, New York, NY, USA) was used to determine the LDH leakage in cell culture supernatants derived from each well housing the spheroids, across various experimental conditions. For each experiment on the 18th day post-isolation, 100 µL of cell culture supernatants per well (total of 4 replicates per fish, 1 from each plate/condition), previously centrifuged at 1500 rpm (0.2 rcf) for 5 min, were transferred to 96-well microplates (351172, non-tissue culture treated sterile plate with a flat bottom and low evaporation lid, Falcon, Corning, New York, NY, USA). Background controls were also included by adding 100 µL of fresh culture medium (*n* = 8 wells). Then, all wells received 100 μL of working solution, and the plate was incubated for 30 min at room temperature (±20 °C), protected from light. Absorbances were measured at 490 nm after stopping the reaction in a Multiskan^TM^ GO microplate spectrophotometer (Thermo Scientific, Vantaa, Finland). Background subtraction was performed, and data were plotted for each condition.

### 2.6. AlamarBlue™ HS Cell Viability Reagent Assay

A total of 8 spheroids (total of 4 replicates per fish, 1 from each plate per condition) were transferred individually to a 96-well microplate (351172, non-tissue culture treated sterile plate with a flat bottom and low evaporation lid, Falcon, Corning, New York, NY, USA). After transfer, 90 µL of the fresh culture medium was added to each well, followed by 10 μL of the AlamarBlue™ HS Cell Viability Reagent (A50101, Invitrogen, Thermo Fisher Scientific, Life Technologies Corporation, Eugene, OR, USA). Blanks (*n* = 8 wells) were performed using the same amount of the corresponding medium but without spheroids. The plates were incubated at 18 °C for 24 h, protected from light, and at constant agitation (~100 rpm) (IKA^®^ MTS 2/4 digital microtiter shaker). The fluorescence was read at 550 nm and 588 nm (excitation and emission lengths, respectively) in a microplate Biotek Synergy™ HTX multimode reader (Agilent, Santa Clara, CA, USA) with the software Gen5 version 3.05.11 (Agilent, Santa Clara, CA, USA). The fluorescence values of each sample were adjusted by blank subtraction and plotted for each condition.

### 2.7. Spheroids Morphology

For each experiment on the 18th day, spheroids (*n* = 6 per condition per fish) were transferred individually into 1.5 mL microtubes. The fixation was conducted using 500 µL of 10% buffered formalin (Epredia, Breda, The Netherlands) at room temperature. After 24 h, the fixative was changed to 70% ethanol. Richard-Allan Scientific HistoGel (HG-4000, Epredia, Breda, The Netherlands) was used to embed the spheroids. Dehydration, clearing, and paraffin impregnation were carried out over 12 h in an automatic processor (TP 1020, Leica Biosystems, Wetzlar, Germany). Posteriorly, the samples were embedded in paraffin (Histoplast, Epredia, Breda, The Netherlands) using an embedding station (EG1140C, Leica Biosystems, Wetzlar, Germany). After embedding, 1 spheroid/fish per exposure condition was sectioned with a thickness of 3 μm in a fully automated rotary microtome (RM2255, Leica Biosystems, Wetzlar, Germany). Sections were stained with hematoxylin and eosin (H&E), and their visualization and photographs were obtained using a light microscope (BX50, Olympus, Tokyo, Japan) coupled with a digital camera (EP50, Olympus, Tokyo, Japan).

### 2.8. RNA Extraction and cDNA Synthesis

One pool of spheroids per condition was obtained on the 18th day post-isolation for each independent experiment. The minimum number of brown trout PHS required to obtain the pellet was previously optimized [21]. Spheroids were transferred into 1.5 mL microtubes, centrifuged at 1500 rpm for 5 min, and the pellets were snap-frozen in liquid nitrogen and stored at –80 °C. Total RNA was extracted with an illustra^TM^ RNAspin Mini RNA Isolation Kit (GE Healthcare, Chicago, IL, USA), which involves a step of treating with DNase I to prevent contamination from genomic DNA. RNA quantification and purity were determined using a Multiskan^TM^ GO microplate spectrophotometer (Thermo Scientific, Vantaa, Finland) with a μDrop™ Plate and a SkanIt Microplate Reader software version 4.1. The cDNA synthesis was performed using the iScript™ Reverse Transcription Supermix kit (Bio-Rad, Hercules, CA, USA), using 300 ng of total RNA for a total volume of 20 μL.

### 2.9. Quantitative Real-Time Polymerase Chain Reaction (RT-qPCR)

The CFX Connect real-time PCR detection system and CFX Manager software version 3.1 (Bio-Rad, Hercules, CA, USA) were used for RT-qPCR. Reactions included 5 μL of cDNA (diluted 1:5), 10 μL of iQ™ SYBR^®^ Green Supermix (Bio-Rad, Hercules, CA, USA), and 200 nM of each primer (total volume of 20 µL). Duplicates of cDNA samples and no-template controls were always included in each analysis. A melt curve was used to assess the product’s specificity.

For the relative gene quantification, the Pfaffl method was used [29], and the normalization was performed using the geometric mean of the two most stable reference genes (*glyceraldehyde-3-phosphate dehydrogenase*—*gapdh* and *ribosomal protein l8*—*rpl8*), based on the NormFinder algorithm [30]. Target genes included *acyl-CoA long chain synthetase 1* (*Acsl1*), *apolipoprotein AI* (*ApoAI*), *catalase* (*Cat*), *estrogen receptor α* (*ERα*), *estrogen receptor β* (*ERβ*), *fatty acid binding protein 1* (*Fabp1*), *urate oxidase* (*Uox*), *vitellogenin A* (*VtgA*), *zona pellucida glycoprotein 2.5* (*ZP2.5*), and *zona pellucida glycoprotein 3a.2* (*ZP3a.2*). The conditions and primer sequences are in Table 1.

### 2.10. Immunohistochemistry

The histological slides were deparaffinized with xylene (2 × 10 min) and hydrated using a series of ethanol (100%, 95%, and 70%). Then, two antigen retrieval protocols were followed according to the specific antibody. For Vtg, the sections were immersed in Tris/EDTA, pH 9.0, and heated for 15 min in a microwave (700 W) after boiling. For ZP, the antigen retrieval was performed by immersing the slides in citrate buffer 0.01 M, pH 6, and heating them in a pressure cooker for 3 min after reaching the maximum pressure. All slides were then left to cool to room temperature. Next, a solution of 3% hydrogen peroxide (Merck KGaA, Darmstadt, Germany) in methanol was used to block the endogenous peroxidase over 10 min. The following protocol steps followed the NovoLink™ Max Polymer Detection Kit (RE7280-K, Leica Biosystems, Newcastle, UK) recommendations. For the Vtg and ZP immunohistochemistry procedures, the sections were incubated for 2 h (at ±20 °C) in a humidified chamber with a polyclonal rabbit anti-Arctic char Vtg, PO-1 (V01409201, Biosense Laboratories AS, Bergen, Norway) antibody, and a polyclonal rabbit anti-salmon zona radiata protein, O-146 antibody (Z03402202, Biosense Laboratories AS, Bergen, Norway), respectively. The Vtg and ZP antibodies were used at a dilution of 1:2500 and 1:3000, respectively, in phosphate-buffered saline (PBS) with 5% bovine serum albumin (BSA) (NZYTech, Lisbon, Portugal), as previously implemented in brown trout [35]. Negative controls were incubated in PBS with BSA 5% (NZYTech, Lisbon, Portugal), and positive controls were liver sections from a mature female brown trout. The 3,3′-diaminobenzidine (DAB) was used as chromogen, and counterstaining was achieved with Mayer’s hematoxylin (Merck KGaA, Darmstadt, Germany) for 1 min. The slides were dehydrated in ethanol, cleared in xylene, and mounted with Q Path^®^ Coverquick 2000 media (VWR Chemicals, Fontenay-sous-Bois, France). A light microscope (BX50, Olympus, Tokyo, Japan) and a digital camera (EP50, Olympus, Tokyo, Japan) were used to photograph the spheroid sections.

### 2.11. Statistical Analyses

Descriptive and inferential statistics and graphs were created using Past 3 software version 3.25 [36] and the GraphPad Prism version 8.0.1, respectively. Shapiro–Wilk and Levene’s tests checked the assumptions of normality and homogeneity of variance of data sets, respectively. A one-way analysis of variance (ANOVA) was followed by Tuckey’s pairwise comparison post-hoc test. The non-parametric Kruskal–Wallis ANOVA and the Mann–Whitney pairwise comparison post-hoc test with sequential Bonferroni corrections were used in a few cases where the mentioned assumptions were not verified, even after data transformation. The differences were considered significant for *p* < 0.05.

## 3. Results

### 3.1. Morphological Parameters

**Figure 1:**
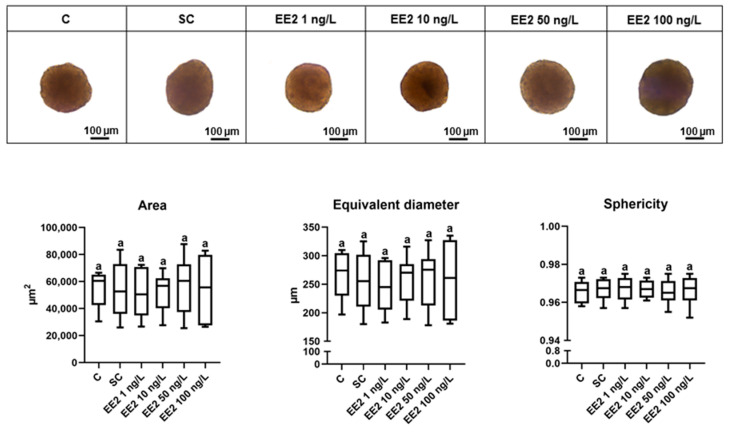
Light microscopy images, area, equivalent diameter, and sphericity of brown trout PHS (total of 6 independent fish, *n* = 30 spheroids per condition) after 6 days exposure (12th to 18th post-isolation day) to distinct conditions: C—control (supplemented DMEM/F-12 medium), SC—solvent control (0.1% ethanol in supplemented DMEM/F-12 medium), EE2 1 ng/L—1 ng/L of EE2 in supplemented DMEM/F-12 medium, EE2 10 ng/L—10 ng/L of EE2 in supplemented DMEM/F-12 medium, EE2 50 ng/L—50 ng/L of EE2 in supplemented DMEM/F-12 medium, and EE2 100 ng/L—100 ng/L of EE2 in supplemented DMEM/F-12 medium. Data correspond to median, minimum, maximum, 25th, and 75th percentiles. Common lower-case letters indicate no significant differences (*p* > 0.05) between conditions.

Bright-field images showed compact spheroids in all conditions with well-defined limits (Figure 1). For all experimental conditions, the area, equivalent diameter, and sphericity of the spheroids were not significantly influenced by EE2 exposures (Figure 1).

### 3.2. Viability—LDH and Resazurin Assays

**Figure 2:**
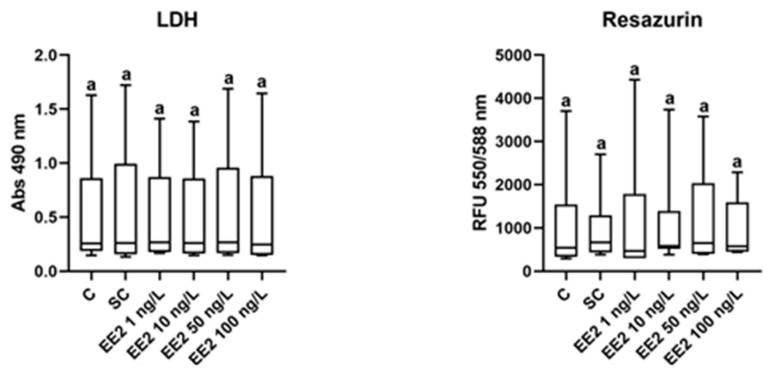
LDH and resazurin assays from brown trout PHS (total of 6 independent fish, *n* = 8 spheroids per condition) after 6 days of exposure (12th to 18th post-isolation day) to distinct conditions: C—control (supplemented DMEM/F-12 medium), SC—solvent control (0.1% ethanol in supplemented DMEM/F-12 medium), EE2 1 ng/L—1 ng/L of EE2 in supplemented DMEM/F-12 medium, EE2 10 ng/L—10 ng/L of EE2 in supplemented DMEM/F-12 medium, EE2 50 ng/L—50 ng/L of EE2 in supplemented DMEM/F-12 medium, and EE2 100 ng/L—100 ng/L of EE2 in supplemented DMEM/F-12 medium. Absorbance values at 490 nm and relative fluorescence units (RFU 550/588 nm) were plotted against each condition. Data correspond to median, minimum, maximum, 25th, and 75th percentiles. Common lower-case letters indicate no significant differences (*p* > 0.05) between conditions.

The LDH and resazurin assays did not evidence significant differences between exposure conditions, as shown in Figure 2.

### 3.3. Morphology

**Figure 3:**
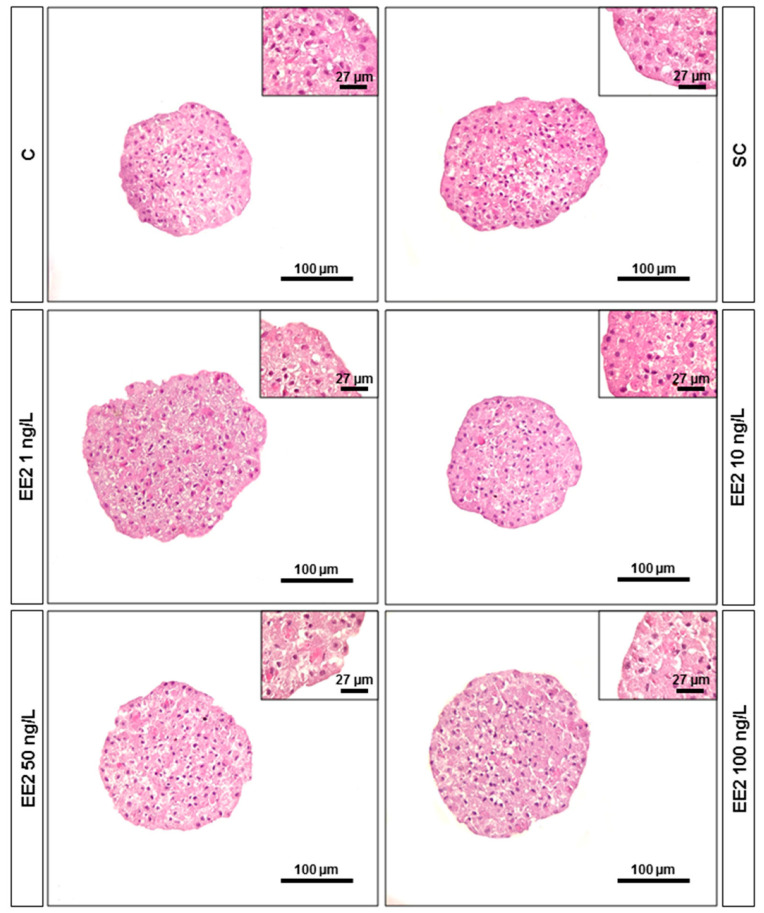
Hematoxylin and eosin (H&E) stained histological sections of brown trout PHS (total of 6 independent fish, *n* = 6 spheroids per condition) after 6 days exposure (12th to 18th post-isolation day) to distinct conditions. C—control (supplemented DMEM/F-12 medium); SC—solvent control (0.1% ethanol in supplemented DMEM/F-12 medium), EE2 1 ng/L—1 ng/L of EE2 in supplemented DMEM/F-12 medium, EE2 10 ng/L—10 ng/L of EE2 in supplemented DMEM/F-12 medium, EE2 50 ng/L—50 ng/L of EE2 in supplemented DMEM/F-12 medium, and EE2 100 ng/L—100 ng/L of EE2 in supplemented DMEM/F-12 medium.

No evident alterations were noted in the general structure of PHS between exposure conditions (Figure 3). Overall, spheroids showed a spherical/elliptical shape in all conditions. Hepatocytes had well-defined cellular limits and an intact nucleus (Figure 3).

### 3.4. RT-qPCR

**Figure 4:**
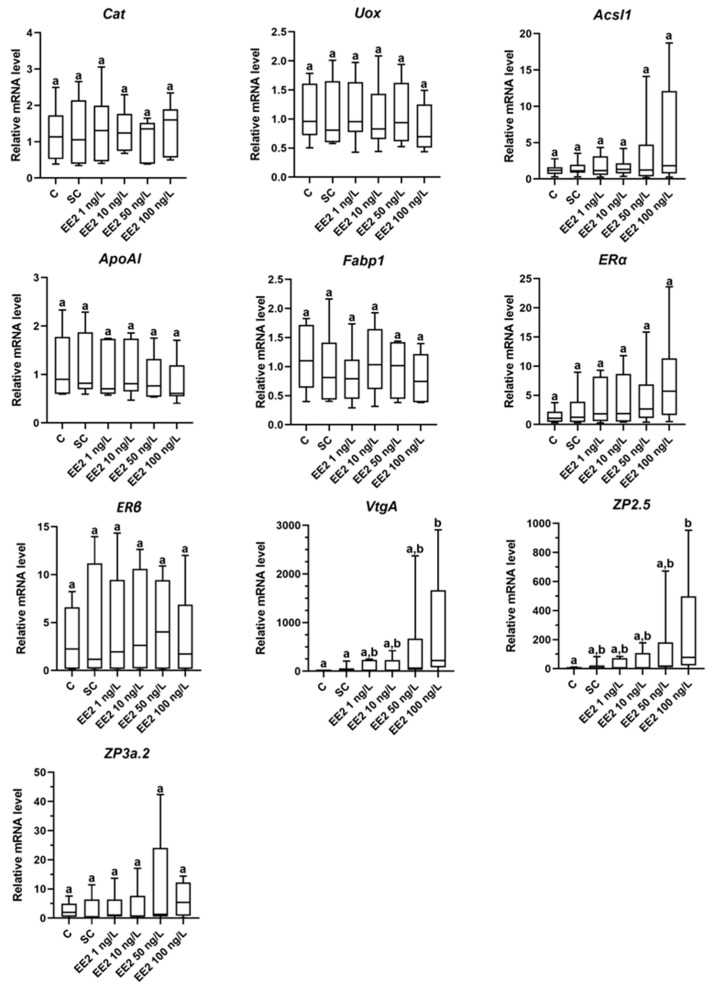
Relative mRNA levels of peroxisomal (*Cat* and *Uox*), lipid (*Acsl1*, *ApoAI* and *Fabp1*), and estrogen-related target (*ERα*, *ERβ*, *VtgA*, *ZP2.5* and *ZP3a.2*) genes of brown trout PHS (total of 6 independent fish, *n* = 1 pools of spheroids per condition) after 6 days of exposure (12th to 18th post-isolation day) to distinct conditions: C—control (supplemented DMEM/F-12 medium), SC—solvent control (0.1% ethanol in supplemented DMEM/F-12 medium), EE2 1 ng/L—1 ng/L of EE2 in supplemented DMEM/F-12 medium, EE2 10 ng/L—10 ng/L of EE2 in supplemented DMEM/F-12 medium, EE2 50 ng/L—50 ng/L of EE2 in supplemented DMEM/F-12 medium, and EE2 100 ng/L—100 ng/L of EE2 in supplemented DMEM/F-12 medium. Data correspond to median, minimum, maximum, 25th, and 75th percentiles. Conditions not showing common letters differ significantly (a vs. b, *p* < 0.05). Significant differences (*p* < 0.05) between groups are represented by different letters. *Cat—catalase*; *Uox*—*urate oxidase*; *Acsl1*—*acyl-CoA long chain synthetase 1*; *ApoAI*—*apolipoprotein AI*; *Fabp1*– *fatty acid binding protein 1*; *ERα*—*estrogen receptor alpha*; *ERβ*—*estrogen receptor beta*; *VtgA*—*vitellogenin A*; *ZP2.5*—*zona pellucida glycoprotein 2.5*; and *ZP3a.2*—*zona pellucida glycoprotein 3.2*.

The lipid metabolism- and peroxisome-related genes were not significantly influenced by EE2 exposure (Figure 4). Regarding the direct estrogen-related genes, the *ZP3a.2*, *ERα*, and *ERβ* showed stable mRNA levels, while *VtgA* and *ZP2.5* expressions were dose-responsive and significantly upregulated at the highest concentration (100 ng/L) (Figure 4).

### 3.5. Immunohistochemistry

**Figure 5:**
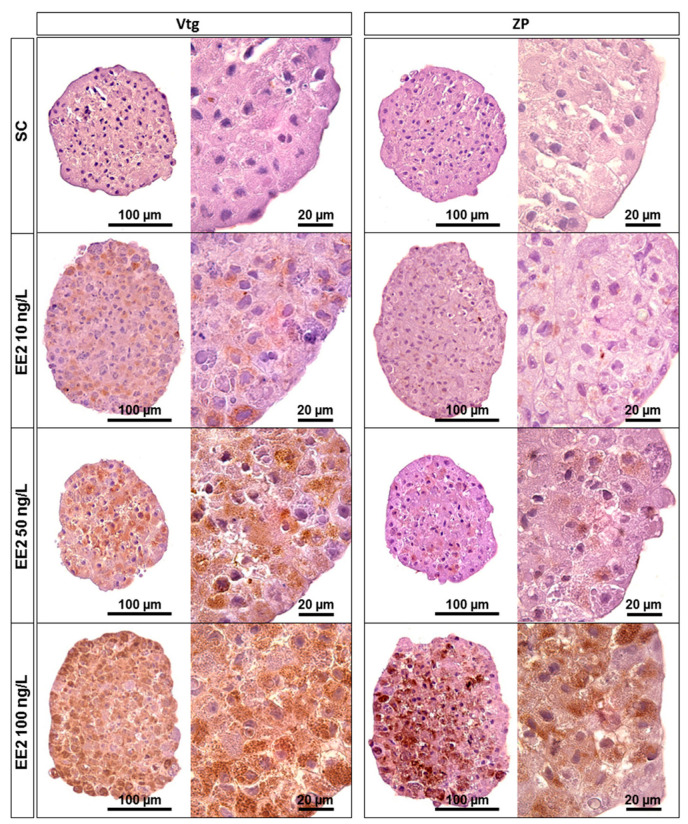
Immunohistochemistry staining for Vtg and ZP on 18-day-old spheroids from brown trout PHS after 6 days of exposure (12th to 18th day post-isolation) to distinct conditions: SC—solvent control (supplemented DMEM/F-12 medium), EE2 10 ng/L—10 ng/L of EE2 in supplemented DMEM/F-12 medium, EE2 50 ng/L—50 ng/L of EE2 in supplemented DMEM/F-12 medium, and EE2 100 ng/L—100 ng/L of EE2 in supplemented DMEM/F-12 medium. Positive immunolabelling corresponds to a brown colour signal.

Overall, for both antibodies used, the C and SC conditions showed no significant immunostaining (Figure 5), whereas EE2 exposure resulted in positive labelling, which was classified into three levels of intensity (in particular for Vtg): low, moderate, and strong. Vtg and ZP immunostaining were both cytoplasmic and varied between diffuse and a granular pattern homogeneously distributed throughout the spheroids. Despite inter-animal variability, the overall pattern showed weaker immunostaining signals in spheroids exposed to EE2 concentrations of 1 ng/L and 10 ng/L (Figure 5). Moderate to strong immunolabelling was noticed for EE2 at 50 ng/L and 100 ng/L (Figure 5), respectively. The pattern obtained with both antibodies was well-defined, especially for the 100 ng/L condition. Negative controls did not show immunostaining.

## 4. Discussion

Like in other research areas, the practice in ecotoxicology has been to limit the number of animals killed. In mammals, 3D hepatocyte cultures remain viable with morphological and functional capacities closer to in vivo liver and over extended periods more than the ones reported in two-dimensional (2D) cultures [18].

Hence, 3D cultures have recently been explored for developing and characterising new fish models [37,38,39]. In this context, environmentally realistic conditions must be evaluated in 3D fish models before these can be adopted as accurate alternatives to in vivo studies. As such, the present study is a step toward determining the potential of using fish 3D models by testing environmental exposure concentrations. Using a previously developed spheroid model of primary brown trout hepatocytes, this study investigated the effects of EE2 at low, medium, and high environmental concentrations on estrogen-related targets.

EE2 environmental levels are variable, ranging from 0.002 ng/L [40] to 45 µg/L [41]. The goal here was to test four concentrations of EE2, three of which were within the range of levels most frequently detected in the environment, as well as a concentration of 100 ng/L; although above the average, environmental monitoring also found such levels [41,42,43].

The morphological parameters (area, equivalent diameter, and sphericity) of 18-day-old brown trout PHS were not influenced by any EE2 concentration. Compact spheroids were observed in all conditions, which indicates that these EE2 levels did not compromise the cell–cell interactions and, consequently, the 3D structure of spheroids. Viable and metabolically competent brown trout PHS were observed in all conditions and resembled the biometric and morphological characteristics previously described for these spheroids with the same days in culture [22].

The genes selected here to evaluate the effect of EE2 were based on in vivo and in vitro assays, using liver or 2D hepatocytes isolated from brown trout, respectively [6,31,35]. In these studies, the target genes associated with various pathways, including estrogenic, peroxisome, and lipid-related pathways, exhibited estrogen-specific responses.

Here, there were no significant differences in the mRNA levels of the selected peroxisomal and lipid metabolism-related genes after 6 days of exposure to EE2. Lipid-related pathways are frequently listed as estrogen-responsive in fish [3,44,45]. Therefore, the absence of changes in the genes tested here may be related to the model, the doses, exposure time, and/or selected targets. For instance, in zebrafish liver, the expression of *ApoAI* was significantly downregulated in response to 10 ng/L of EE2 for 21 days [46]. According to Madureira et al. [6], the same peroxisomal (*Cat* and *Uox*) and lipid (*Fabp1* and *ApoAI*) target genes in brown trout liver showed a decrease or an increase (*Acsl1*) in gene expression after 28 days of in vivo exposure to EE2 (50 μg/L) [6]. However, it should be noted that in the latter example, the selected concentration is approximately 5000-fold times higher than the usual environmental range (using a concentration of 10 ng/L as reference). A distinct profile was found regarding the expression of the *VtgA* and *ZP2.5* genes, both widely accepted as estrogenic markers [47]. In this study, the *VtgA* and *ZP2.5* mRNA levels were significantly higher after exposure to EE2 at 100 ng/L compared to the other groups. An increasing trend was evident following exposure to 50 ng/L of EE2, although statistical significance was not proved. Data from in vivo EE2 experiments with different fish species support our results with brown trout PHS. For instance, increases in *Vtg* gene expression were observed in rainbow trout and male medaka (*Oryzias latipes*) after water exposure to 125 ng/L and 100 ng/L of EE2 over 61 days [7] and 4 weeks [48], respectively. Uren Webster et al. [33] found a significant upregulation of *vtg1*, *ZP2.5*, and *ZP3a.2* genes after 4 days of water treatment with 34.4 ng/L of E2. Interestingly, *ZP2.5* transcript was more responsive to E2 than *ZP3a.2*, which aligns with our results. Also, in brown trout, low doses of EE2 (3 ng/L) for 21 days caused a *Vtg* mRNA induction in vivo [5], but this did not happen in PHS, at least for the exposure time tested here. Evidence linked *Vtg* induction in trout to *ER* expression [49], but the *ERα* and *ERβ* mRNA levels did not change significantly in the present study. Mortensen and Arukwe [50] showed that liver *ERα* and *ERβ* mRNA levels significantly decreased, while *Vtg* and *ZP* increased at day 3 after EE2 exposures (50 ng/L) in juvenile Atlantic salmon. It was suggested by the authors that basal *ER* levels may be sufficient to trigger *Vtg* and *ZP* gene inductions, which may justify here the steady *ER* expression with increases of *VtgA* and *ZP2.5* mRNA levels. Further, prior studies demonstrated a peak induction of *ERα* mRNA levels in less than 48 h after EE2 exposure in rainbow trout [7]. As a result, we cannot rule out the possibility that we did not assess (at least) *ERα* expression at its maximum induction time.

In vitro studies with hepatocyte cell cultures also corroborate the *VtgA* and *ZP2.5* gene expression patterns obtained in this study. In rainbow trout primary hepatocytes exposed to EE2 for 48 h, there was a dose–effect concentration on the upregulation of *vtg1*, *zrp3*, and *zrp4* levels for concentrations above 88.9 ng/L (0.3 nM) [9]. In fish hepatocyte spheroid cultures, estrogenic stimulation has also been tested but, so far, in unrealistic environmental concentrations [25,27,51,52]. For instance, according to Pelissero et al. [52], EE2 highly increased the Vtg concentrations in the culture medium of rainbow trout hepatocyte aggregates at the 296,400—29,640,000 ng/L (1000–100,000 nM) range, although minimal induction was measured at 296.4 ng/L (1 nM). Flouriot et al. [27] described that the *Vtg* mRNA induction was caused by exposure to 272,380 ng/L (1 µM) of 17β-estradiol (E2) during 24 h in male rainbow trout hepatocyte spheroids. Also, in the zebrafish liver (ZFL) cell line, the *Vtg5* mRNA expression was upregulated after exposure to E2 (1 nM = 272.38 ng/L) [25].

Within the present study, EE2 also caused a specific induction of Vtg and ZP protein expression, as already evidenced in the liver of Atlantic salmon exposed to other estrogenic compounds [53,54]. The positive control labelling for Vtg and ZP protein was cytoplasmic, as previously reported for both antibodies in brown trout hepatocytes [35]. Here, immunohistochemistry corroborated gene expression, showing a consistent increase in Vtg and ZP proteins, particularly after exposure to 100 ng/L of EE2. Immunolabelling was weaker and variable across the fish for lower EE2 concentrations (1 and 10 ng/L). The effect concentration that caused a 10-fold Vtg protein induction in rainbow trout hepatocytes was 71.7 ng/L (2.42 × 10^−10^ mol/L) [8], which aligns with the data obtained here.

## 5. Conclusions

The present study revealed the applicability, sensitivity, and specific responsiveness to EE2 of primary brown trout hepatocyte spheroids under distinct environmentally relevant levels. At 100 ng/L, remarkable effects were detected, particularly the stimulation of *Vtg* and *ZP* genes and protein expression. Lipid target genes did not change, even at the higher EE2 concentration, under the tested conditions. Given the published evidence from in vivo and 2D in vitro research, the model used here could serve as an alternate diagnostic tool for uncovering some effects arising from exposure to estrogen-disrupting compounds. However, further studies should be considered to disclose the EE2 effects at environmentally relevant levels in brown trout PHS and its applicability as an alternative model in ecotoxicology.

## Figures and Tables

**Table 1:** Primer sequences, annealing temperature (AT), and efficiencies (E).

Gene	Abbreviation	Primer Sequences	AT (°C)	E (%)	References
*Acyl-CoA long chain synthetase 1*	*Acsl1*	F: 5′-CGACCAAGCCGCTATCTC-3′ R: 5′-CCAACAGCCTCCACATCC-3′	55.0	97.8	[6]
*Apolipoprotein AI*	*ApoAI*	F: 5′-ATGAAATTCCTGGCTCTTG-3′ R: 5′-TACTCTTTGAACTCTGTGTC-3′	55.0	89.9	[31]
*Catalase*	*Cat*	F: 5′-CACTGATGAGGGCAACTGGG-3′ R: 5′-CTTGAAGTGGAACTTGCAG-3′	58.0	91.4	[32]
*Estrogen receptor α*	*ERα*	F: 5′-GACATGCTCCTGGCCACTGT-3′ R: 5′-TGGCTTTGAGGCACACAAAC-3′	61.6	91.2	[5]
*Estrogen receptor β*	*ERβ*	F: 5′-TGTGGACCTGTGCCTGTTC-3′ R: 5′-ACATGAGCCCTAGCATCAGC-3′	66.5	103.3	[5]
*Fatty acid binding protein 1*	*Fabp1*	F: 5′-GTCCGTCACCAACTCCTTC-3′ R: 5′-GCGTCTCAACCATCTCTCC-3′	57.0	97.7	[31]
*Urate oxidase*	*Uox*	F: 5′-CTTCCGAGACCGCTTCAC-3′ R: 5′-CATTCTGGACCTTGTTGTAGC-3′	59.0	90.6	[5]
*Vitellogenin A*	*VtgA*	F: 5′-AACGGTGCTGAATGTCCATAG-3′ R: 5′-ATTGAGATCCTTGCTCTTGGTC-3′	62.9	99.0	[5]
*Zona pellucida glycoprotein 2.5*	*ZP2.5*	F: 5′-ATCAATAACCACAGCCACAATG-3′ R: 5′-ACCAGGGACAGCCAATATG-3′	55.0	99.0	[33]
*Zona pellucida glycoprotein 3a.2*	*ZP3a.2*	F: 5′-AACTACACTCCACTTCATC-3′ R: 5′-CACATCTCCTTCATCTTCA-3′	54.5	101.8	[33]
*Glyceraldehyde-3-phosphate dehydrogenase*	*Gapdh*	F: 5′-CCACCTATGTAGTTGAGTC-3′ R: 5′-ACCTTGAGGGAGTTATCG-3′	55.0	92.8	[34]
*Ribosomal protein l8*	*rpl8*	F: 5′-TCAGCTGAGCTTTCTTGCCAC-3′ R: 5′-AGGACTGAGCTGTTCATTGCG-3′	59.0	93.8	[5]

## Data Availability

Available from the corresponding author upon reasonable request.

## References

[1] Ciślak M., Kruszelnicka I., Zembrzuska J., Ginter-Kramarczyk D. (2023). Estrogen pollution of the european aquatic environment: A critical review. Water Res..

[2] Sun S.X., Wu J.L., Lv H.B., Zhang H.Y., Zhang J., Limbu S.M., Qiao F., Chen L.Q., Yang Y., Zhang M.L. (2020). Environmental estrogen exposure converts lipid metabolism in male fish to a female pattern mediated by AMPK and mTOR signaling pathways. J. Hazard. Mater..

[3] Voisin A.S., Kültz D., Silvestre F. (2019). Early-life exposure to the endocrine disruptor 17-α-ethinylestradiol induces delayed effects in adult brain, liver and ovotestis proteomes of a self-fertilizing fish. J. Proteom..

[4] Goundadkar B.B., Katti P. (2017). Environmental estrogen(s) induced swimming behavioural alterations in adult zebrafish (*Danio rerio*). Environ. Toxicol. Pharmacol..

[5] Körner O., Kohno S., Schönenberger R., Suter M.J., Knauer K., Guillette L.J., Burkhardt-Holm P. (2008). Water temperature and concomitant waterborne ethinylestradiol exposure affects the vitellogenin expression in juvenile brown trout (*Salmo trutta*). Aquat. Toxicol..

[6] Madureira T.V., Malhão F., Simões T., Pinheiro I., Lopes C., Gonçalves J.F., Urbatzka R., Castro LF C., Lemos MF L., Rocha E. (2018). Sex-steroids and hypolipidemic chemicals impacts on brown trout lipid and peroxisome signaling—Molecular, biochemical and morphological insights. Comp. Biochem. Physiol. Part C Toxicol. Pharmacol..

[7] Skillman A.D., Nagler J.J., Hook S.E., Small J.A., Schultz I.R. (2006). Dynamics of 17alpha-ethynylestradiol exposure in rainbow trout (*Oncorhynchus mykiss*): Absorption, tissue distribution, and hepatic gene expression pattern. Environ. Toxicol. Chem..

[8] Hultman M.T., Rundberget J.T., Tollefsen K.E. (2015). Evaluation of the sensitivity, responsiveness and reproducibility of primary rainbow trout hepatocyte vitellogenin expression as a screening assay for estrogen mimics. Aquat. Toxicol..

[9] Hultman M.T., Song Y., Tollefsen K.E. (2015). 17α-Ethinylestradiol (EE2) effect on global gene expression in primary rainbow trout (*Oncorhynchus mykiss*) hepatocytes. Aquat. Toxicol..

[10] Finne E.F., Cooper G.A., Koop B.F., Hylland K., Tollefsen K.E. (2007). Toxicogenomic responses in rainbow trout (Oncorhynchus mykiss) hepatocytes exposed to model chemicals and a synthetic mixture. Aquat. Toxicol..

[11] Madureira T.V., Malhão F., Pinheiro I., Lopes C., Ferreira N., Urbatzka R., Castro L.F., Rocha E. (2015). Estrogenic and anti-estrogenic influences in cultured brown trout hepatocytes: Focus on the expression of some estrogen and peroxisomal related genes and linked phenotypic anchors. Aquat. Toxicol..

[12] Martyniuk C.J., Feswick A., Munkittrick K.R., Dreier D.A., Denslow N.D. (2020). Twenty years of transcriptomics, 17alpha-ethinylestradiol, and fish. Gen. Comp. Endocrinol..

[13] Yadetie F., Zhang X., Hanna E.M., Aranguren-Abadía L., Eide M., Blaser N., Brun M., Jonassen I., Goksøyr A., Karlsen O.A. (2018). RNA-Seq analysis of transcriptome responses in Atlantic cod (*Gadus morhua*) precision-cut liver slices exposed to benzo[a]pyrene and 17α-ethynylestradiol. Aquatic Toxicology.

[14] Mcgovarin S., Nishikawa J., Metcalfe C.D. (2022). Vitellogenin induction in mucus from brook trout (*salvelinus fontinalis*). Bull. Environ. Contam. Toxicol..

[15] Berg A.H., Westerlund L., Olsson P.E. (2004). Regulation of arctic char (*Salvelinus alpinus*) egg shell proteins and vitellogenin during reproduction and in response to 17beta-estradiol and cortisol. Gen. Comp. Endocrinol..

[16] Kozyra M., Johansson I., Nordling Å., Ullah S., Lauschke V.M., Ingelman-Sundberg M. (2018). Human hepatic 3D spheroids as a model for steatosis and insulin resistance. Sci. Rep..

[17] Kanebratt K.P., Janefeldt A., Vilén L., Vildhede A., Samuelsson K., Milton L., Björkbom A., Persson M., Leandersson C., Andersson T.B. (2021). Primary human hepatocyte spheroid model as a 3D in vitro platform for metabolism studies. J. Pharm. Sci..

[18] Bell C.C., Hendriks D.F., Moro S.M., Ellis E., Walsh J., Renblom A., Fredriksson P.L., Dankers A.C., Jacobs F., Snoeys J. (2016). Characterization of primary human hepatocyte spheroids as a model system for drug-induced liver injury, liver function and disease. Sci. Rep..

[19] Foster A.J., Chouhan B., Regan S.L., Rollison H., Amberntsson S., Andersson L.C., Srivastava A., Darnell M., Cairns J., Lazic S.E. (2019). Integrated in vitro models for hepatic safety and metabolism: Evaluation of a human Liver-Chip and liver spheroid. Arch. Toxicol..

[20] Song Y., Kim N., Heo J., Shum D., Heo T., Seo H.R. (2024). Inhibition of DNMT3B expression in activated hepatic stellate cells overcomes chemoresistance in the tumor microenvironment of hepatocellular carcinoma. Sci. Rep..

[21] Pereira I.L., Lopes C., Rocha E., Madureira T.V. (2022). Establishing brown trout primary hepatocyte spheroids as a new alternative experimental model–Testing the effects of 5α-dihydrotestosterone on lipid pathways. Aquat. Toxicol..

[22] Alves R.F., Lopes C., Rocha E., Madureira T.V. (2023). A step forward in the characterization of primary brown trout hepatocytic spheroids as experimental models. Animals.

[23] Wang T., Desmet J., Pérez-Albaladejo E., Porte C. (2023). Development of fish liver PLHC-1 spheroids and its applicability to investigate the toxicity of plastic additives. Ecotoxicol. Environ. Saf..

[24] Lammel T., Tsoukatou G., Jellinek J., Sturve J. (2019). Development of threedimensional (3D) spheroid cultures of the continuous rainbow trout liver cell line RTLW1. Ecotoxicol. Environ. Saf..

[25] Park C.G., Ryu C.S., Sung B., Manz A., Kong H., Kim Y.J. (2022). Transcriptomic and physiological analysis of endocrine disrupting chemicals impacts on 3D zebrafish liver cell culture system. Aquat. Toxicol..

[26] Rodd A.L., Messier N.J., Vaslet C.A., Kane A.B. (2017). A 3D fish liver model for aquatic toxicology: Morphological changes and Cyp1a induction in PLHC-1 microtissues after repeated benzo(a)pyrene exposures. Aquat. Toxicol..

[27] Flouriot G., Vaillant C., Salbert G., Pelissero C., Guiraud J.M., Valotaire Y. (1993). Monolayer and aggregate cultures of rainbow trout hepatocytes: Long-term and stable liver-specific expression in aggregates. J. Cell Sci..

[28] Piccinini F. (2015). AnaSP: A software suite for automatic image analysis of multicellular spheroids. Comput. Methods Programs Biomed..

[29] Pfaffl M.W. (2001). A new mathematical model for relative quantification in real-time RTPCR. Nucleic Acids Res..

[30] Andersen C.L., Jensen J.L., Ørntoft T.F. (2004). Normalization of real-time quantitative reverse transcription-PCR data: A model-based variance estimation approach to identify genes suited for normalization, applied to bladder and colon cancer data sets. Cancer Res..

[31] Madureira T.V., Pinheiro I., Malhão F., Lopes C., Urbatzka R., Castro LF C., Rocha E. (2017). Cross-interference of two model peroxisome proliferators in peroxisomal and estrogenic pathways in brown trout hepatocytes. Aquat. Toxicol..

[32] Batista-Pinto C. (2007). Peroxisomes in Brown trout (*Salmo trutta f. fario*): Regulation by Estrogens. Ph.D Thesis.

[33] Uren Webster T.M., Shears J.A., Moore K., Santos E.M. (2015). Identification of conserved hepatic transcriptomic responses to 17β-estradiol using high-throughput sequencing in brown trout. Physiol. Genom..

[34] Madureira T.V., Pinheiro I., De Paula Freire R., Rocha E., Castro L.F., Urbatzka R. (2017). Genome specific PPARαB duplicates in salmonids and insights into estrogenic regulation in brown trout. Comp. Biochem. Physiol. Part B Biochem. Mol. Biol..

[35] Lopes C., Madureira T.V., Gonçalves J.F., Rocha E. (2020). Disruption of classical estrogenic targets in brown trout primary hepatocytes by the model androgens testosterone and dihydrotestosterone. Aquat. Toxicol..

[36] Hammer Ø., Harper D.A., Ryan P.D. (2001). PAST: Paleontological statistics software package for education and data analysis. Palaeontol. Electron..

[37] Alves R.F., Rocha E., Madureira T.V. (2022). Fish hepatocyte spheroids—A powerful (though underexplored) alternative in vitro model to study hepatotoxicity. Comp. Biochem. Physiol. Part C Toxicol. Pharmacol..

[38] Park C.G., Jun I., Lee S., Ryu C.S., Lee S.A., Park J., Han H.S., Park H., Manz A., Shin H. (2022). Integration of bioinspired fibrous strands with 3D spheroids for environmental hazard monitoring. Small.

[39] Langanl M., Dodd N.J., Owen S.F., Purcell W.M., Jackson S.K., Jha A.N. (2016). Direct measurements of oxygen gradients in spheroid culture system using electron parametric resonance oximetry. PLoS ONE.

[40] Avar P., Zrínyi Z., Maász G., Takátsy A., Lovas S., G-Tóth L., Pirger Z. (2016). β-Estradiol and ethinyl-estradiol contamination in the rivers of the Carpathian Basin. Environ. Sci. Pollut. Res. Int..

[41] Griffero L., Alcántara-Durán J., Alonso C., Rodríguez-Gallego L., Moreno-González D., García-Reyes J.F., Molina-Díaz A., Pérez-Parada A. (2019). Basin-scale monitoring and risk assessment of emerging contaminants in South American Atlantic coastal lagoons. Sci. Total Environ..

[42] Valdés M.E., Marino D.J., Wunderlin D.A., Somoza G.M., Ronco A.E., Carriquiriborde P. (2015). Screening concentration of E1, E2 and EE2 in sewage effluents and surface waters of the “Pampas” region and the “Río de la Plata” estuary (Argentina). Bull. Environ. Contam. Toxicol..

[43] Klaic M., Jirsa F. (2022). 17α-Ethinylestradiol (EE2): Concentrations in the environment and methods for wastewater treatment—An update. RSC Adv..

[44] Zhang X., Zhong H., Han Z., Tang Z., Xiao J., Guo Z., Wang F., Luo Y., Zhou Y. (2020). Effects of waterborne exposure to 17β-estradiol on hepatic lipid metabolism genes in tilapia (*Oreochromis niloticus*). Aquac. Rep..

[45] Lourenço T., Rocha E., Gonçalves J.F., Rocha M.J., Madureira T.V. (2024). A proof-of-concept for a hypolipidemic brown trout model. Toxics.

[46] Martyniuk C.J., Gerrie E.R., Popesku J.T., Ekker M., Trudeau V.L. (2007). Microarray analysis in the zebrafish (*Danio rerio*) liver and telencephalon after exposure to low concentration of 17alpha-ethinylestradiol. Aquat. Toxicol..

[47] Celius T., Matthews J.B., Giesy J.P., Zacharewski T.R. (2000). Quantification of rainbow trout (*Oncorhynchus mykiss*) zona radiata and vitellogenin mRNA levels using real-time PCR after in vivo treatment with estradiol-17 beta or alpha-zearalenol. J. Steroid Biochem. Mol. Biol..

[48] Scholz S., Kordes C., Hamann J., Gutzeit H.O. (2004). Induction of vitellogenin in vivo and in vitro in the model teleost medaka (*Oryzias latipes*): Comparison of gene expression and protein levels. Mar. Environ. Res..

[49] Leaños-Castañeda O., Van Der Kraak G. (2007). Functional characterization of estrogen receptor subtypes, ERalpha and ERbeta, mediating vitellogenin production in the liver of rainbow trout. Toxicol. Appl. Pharmacol..

[50] Mortensen A.S., Arukwe A. (2007). Effects of 17alpha-ethynylestradiol on hormonal responses and xenobiotic biotransformation system of Atlantic salmon (*Salmo salar*). Aquat. Toxicol..

[51] Sullivan K.M., Park C.G., Ito J.D., Kandel M., Popescu G., Kim Y.J., Kong H. (2020). Matrix softness-mediated 3D zebrafish hepatocyte modulates response to endocrine disrupting chemicals. Environ. Sci. Technol..

[52] Pelissero C., Flouriot G., Foucher J.L., Bennetau B., Dunoguès J., Le Gac F., Sumpter J.P. (1993). Vitellogenin synthesis in cultured hepatocytes; an in vitro test for the estrogenic potency of chemicals. J. Steroid Biochem. Mol. Biol..

[53] Arukwe A., Røe K. (2008). Molecular and cellular detection of expression of vitellogenin and zona radiata protein in liver and skin of juvenile salmon (*Salmo salar*) exposed to nonylphenol. Cell Tissue Res..

[54] Arukwe A., Nilsen B.M., Berg K., Goksoyr A. (1999). Immunohistochemical analysis of the vitellogenin response in the liver of Atlantic salmon exposed to environmental oestrogens. Biomarkers.

